# *SDHAF2*-Linked Metastatic Paraganglioma: A Case Report with Implications for Counseling and Follow-Up of Pathogenic *SDHAF2* Variant Carriers

**DOI:** 10.1155/2024/2111531

**Published:** 2024-03-20

**Authors:** Monique A. M. de Jong, Eleonora P. M. Corssmit, Jeroen C. Jansen, Thomas P. Potjer, Jean-Pierre L. Bayley, Erik F. Hensen

**Affiliations:** ^1^Department of Otorhinolaryngology and Head and Neck Surgery, Leiden University Medical Center, Leiden, Netherlands; ^2^Department of Endocrinology and Metabolic Diseases, Leiden University Medical Center, Leiden, Netherlands; ^3^Department of Clinical Genetics, Leiden University Medical Center, Leiden, Netherlands; ^4^Department of Human Genetics, Leiden University Medical Center, Leiden, Netherlands

## Abstract

Head and neck paragangliomas are slow growing and highly vascular neuroendocrine tumors. It is currently assumed that *SDHAF2* variants exclusively cause benign and often multicentric head and neck paragangliomas. Here, we present a patient diagnosed with multiple *SDHAF2*-linked head and neck paragangliomas who in addition developed paraganglioma metastases to the lung and spine and a primary or metastatic paraganglioma in the head of the pancreas. During the course of the disease, a range of management strategies were deployed for the different head and neck tumors, including total resections, partial resections, and active surveillance. After identification of the paraganglioma metastases, the patient was treated with lanreotide after which the disease remained stable during the 27 months of follow-up.

## 1. Introduction

Paragangliomas and pheochromocytomas (PPGLs) are slow growing and highly vascular neuroendocrine tumors that occur along the paraganglia pathway of embryologic migration extending from the skull base to the pelvic floor. They can be divided into sympathetic tumors, e.g., pheochromocytomas or extra-adrenal paragangliomas, and parasympathetic tumors, which are primarily found in the head and neck region. The predilection sites in the head and neck region are the carotid bifurcation, along the course of the vagal nerve, the jugular foramen, and the promontory of the middle ear [1, 2]. Head and neck paragangliomas (HNPGL) can cause serious morbidity due to their close relation to major blood vessels and cranial nerves. Most head and neck paragangliomas are benign, but some cause locoregional or distant metastasis. It is estimated that 50% of head and neck paraganglioma patients carry a hereditary predisposition [3].

From the year 2000 onwards, it has become clear that a major cause of paraganglioma is pathogenic variants in genes encoding subunits of succinate dehydrogenase (SDH), a mitochondrial enzyme that plays a key role in the citric acid cycle and the electron transport chain. The first SDH gene linked to paragangliomas was *SDHD*, encoding one of its mitochondrial membrane anchor subunits [4]. Since then, pathogenic variants in the *SDHA*, *SDHB*, and *SDHC* subunits and the *SDHAF2* cofactor have been linked to distinct hereditary paraganglioma syndromes, with different risks of developing associated tumors or metastatic disease, and different risks to family members. Germline pathogenic variants of the *SDHD* gene most frequently cause HNPGLs (in 91% to 98%) but confer a relatively low risk of developing PGL metastases (0–23%) [5]. Compared to *SDHD* variant carriers, patients with pathogenic *SDHB* variants develop metastatic disease more frequently (20–41%) [5]. Metastatic PGLs are seldomly reported in *SDHC* variant carriers.

Previous studies have identified the *SDHAF2* gene (formerly known as PGL2 or SDH5) as the causative gene in a small proportion of patients with head and neck paragangliomas [6]. The largest cluster of *SDHAF2*-linked paraganglioma patients is found in the Netherlands and is known to carry the c.232G > A, p.Gly78Arg variant in exon 2 of the *SDHAF2* gene [7]. The phenotype of this *SDHAF2*-linked family is characterized by the exclusive occurrence of nonmetastatic and often multicentric head and neck paragangliomas. No sympathetic paragangliomas and metastasizing tumors were observed in this study [8]. In 2019, Albattal et al. reported the identification of another *SDHAF2* variant (c.438C > A, p.N146K) in a single patient with a metastatic pheochromocytoma [9]. *SDHAF2* variants show parent-of-origin dependent expression, with tumor development inherited almost exclusively paternally, as seen in *SDHD*-related paraganglioma [7].

Here, we present a patient diagnosed with multiple *SDHAF2*-linked head and neck paragangliomas. Over the course of the disease, the patient developed metastases to the lung and spinal bone, along with a paraganglioma in the head of the pancreas which may be either a primary PGL or a HNPGL metastasis. To our knowledge, this is the first case with documented metastatic disease resulting from a *SDHAF2*-linked paraganglioma reported to date.

## 2. Case Presentation

A 49-year-old man periodically visited our outpatient clinic for endocrinological and otorhinolaryngological evaluation of multiple head and neck paragangliomas, with a strong suspicion of *SDHAF2* variant carrier status.

The patient had presented at the age of 14 years with two slowly progressing swellings on both sides of the neck, diagnosed as bilateral carotid body tumors (CBDs) (no imaging available) (Table 1). The family history revealed a father, aunt, and grandfather in the paternal lineage with multiple head and neck paragangliomas (Table 2). Some of the affected family members had been identified as carriers of the pathogenic c.232G > A, p.Gly78Arg variant in *SDHAF2*, and therefore, the patient was deemed a carrier of the same pathogenic *SDHAF2* variant.

**Figure 1:**
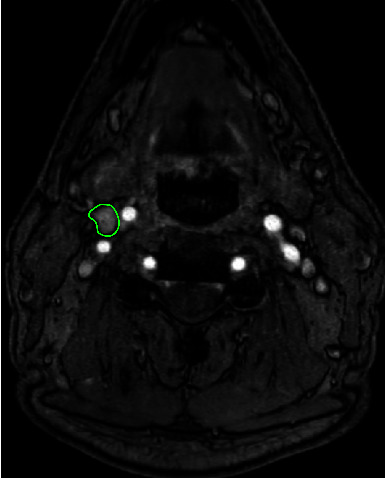
3D TOF with gadolinium MRI showing residual carotic body tumor on the right side (green outline).

**Figure 2:**
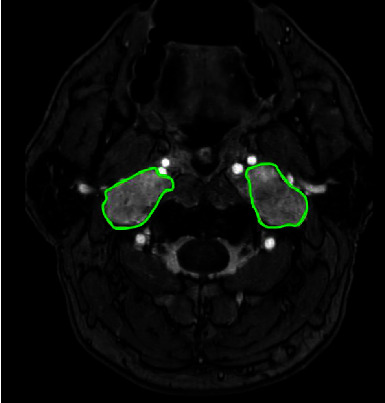
3D TOF with gadolinium MRI showing bilateral vagal paragangliomas (green outlines).

**Figure 3:**
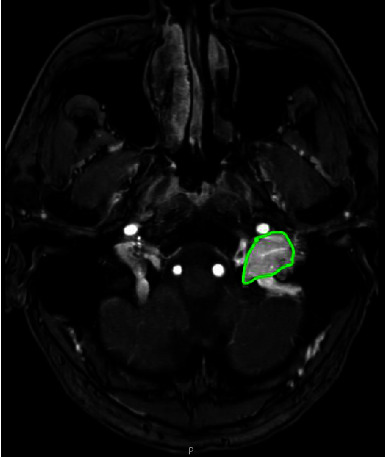
3D TOF with gadolinium MRI showing a jugulotympanic paraganglioma on the left side (green outline).

At the age of 19, both CBTs were surgically removed. Histopathology confirmed the diagnosis of bilateral paraganglioma. Three years after surgery, the patient noticed recurrence of the swelling in the neck on the right, and at the age of 27, recurrence of the right CBT was diagnosed with MR imaging (Figure 1). In addition, the MRI showed bilateral vagal paragangliomas (Figure 2) with the left-sided tumor slowly progressive over time. In the following years, paresis of the right vocal cord developed, accompanied by hoarseness that was acceptable for the patient. At the age of 34, the patient was diagnosed with an additional jugulotympanic paraganglioma on the left side (Figure 3), which was partly surgically removed 16 years later because of troublesome pulsatile tinnitus. This procedure was uncomplicated and resulted in the satisfactory resolution of the tinnitus. No clinically relevant catecholamine excess was found during the course of the disease (Table 3).

**Figure 4:**
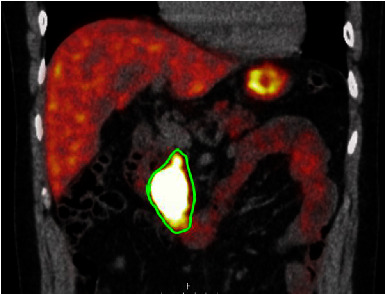
PET-CT 68Ga-DOTATATE scan showing a 3 cm lesion in the head of the pancreas (green outline).

**Figure 5:**
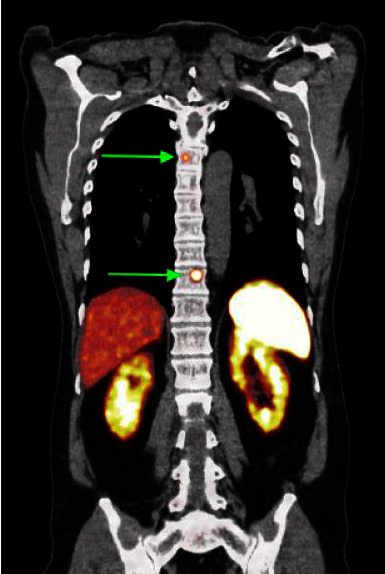
PET-CT 68Ga-DOTATATE scan showing paravertebral lesions (arrows).

At the age of 49, a whole body MRI was performed in a clinical screening context. A new solid lesion of 3 cm at the head of the pancreas was identified. A subsequent 68Ga-DOTATATE scan showed increased uptake in this lesion and revealed additional DOTATATE-avid lesions in the thorax: two suspected noduli in the right lung and multiple paravertebral spinal bone lesions (Figures 4 and 5). No other primary sympathetic paragangliomas or pheochromocytomas were found in the abdomen or thorax. A transpedicular biopsy of the left Th10 showed a localization of paraganglioma within the spinal bone. Cytology of the head of the pancreas also showed paraganglioma. Both specimens showed negative SDHB immunohistochemistry. IHC stainings were performed on 4-5 *μ*m tumor sections of FFPE blocks using a 1/500 dilution of the SDHB polyclonal antibody HPA002868 (Atlas, Stockholm, Sweden) using standard immunohistochemical techniques. These findings confirmed the diagnosis of metastatic *SDHx*-linked paraganglioma. DNA testing of affected family members had already identified the pathogenic c.232G > A p.(Gly78Arg) variant in exon 2 of the *SDHAF2* gene, and this variant was confirmed in the current patient using standard Sanger sequencing. Because of the multicentric and metastatic disease, systemic treatment was started with lanreotide 120 mg daily. The disease has remained stable according to RECIST 1.1 during the 27 months of follow-up, both at the primary and metastatic sites.

## 3. Discussion

The current case shows that, contrary to previous reports, the c.232G > A p.(Gly78Arg) variant in exon 2 of the *SDHAF2* gene does not exclusively cause nonmetastatic head and neck paragangliomas but may also lead to metastatic disease. In the current patient, the lesions outside of the head and neck region may all represent metastases from the parasympathetic head and neck paragangliomas. Alternatively, the lesion in the pancreas may be a primary PPGL, and the metastatic lesions to the bone and possibly the lung may stem from this sympathetic PPGL. As primary and metastatic paraganglioma tissue cannot be reliably differentiated by histopathology, there is no way to reject or confirm one of these scenarios with certainty. Either way, this report describes a unique case, the first with documented *SDHAF2*-linked metastatic parasympathetic or extra-adrenal paraganglioma.

The fact that *SDHAF2*-linked paragangliomas are not always benign and that *SDHAF2*-linked disease is not confined to the head and neck region has implications for the counseling and follow-up of carriers of pathogenic *SDHAF2* variants. Because metastatic and/or sympathetic paragangliomas may require proactive management, whole body tumor screening is warranted, as in carriers of other pathogenic *SDHx* gene variants.

Currently, whole-body MRI screening is a frequently used tool for the clinical screening of *SDHx* variant carriers. However, Janssen et al. showed that the detection rate of 68Ga-DOTATATE PET/CT in SDHB-related metastatic paragangliomas and pheochromocytomas is higher (98.6%) than CT/MRI (84.8%) [10]. According to the international consensus on initial screening and follow-up of asymptomatic *SDHx* mutation carriers also, FDG PET CT is a feasible screening tool [11]. Indeed, in the current patient, more lesions were identified with a 68Ga-DOTATATE scan compared to the whole-body MRI. Like in other *SDHx* pathogenic variants, 68Ga-DOTATATE PET/CT (when available) should be considered in the surveillance protocol of *SDHAF2* carriers too [11]. Implementing this modality may reveal more sympathetic paragangliomas and metastases in the future and show that *SDHAF2*-linked metastatic disease is currently underreported.

Treatment of paraganglioma mostly depends on the location of the tumor, the occurrence of catecholamine hypersecretion, and multicentricity. In nonmetastatic, nonsecreting head and neck paragangliomas, surgical intervention is generally performed if the morbidity caused by the intervention is outweighed by the potential for relief of complaints or the avoidance of more serious disease. Other management options include active surveillance, radiotherapy (with photons or protons), and sometimes partial resection for symptom control. Surgery is potentially curative and may prevent future metastatic disease; however, total resection of the tumor may not be feasible or opportune due to tumor extension and involvement of cranial nerves or critical blood supply to the head and neck region. As the risk of metastases is generally low (and was deemed absent in *SDHAF2*-linked patients), active surveillance may be a viable option. Radiotherapy may be a good alternative for single tumors and may stop tumor progression; however, it is as of yet unclear whether radiotherapy prevents future metastatic disease. Especially in metastatic paraganglioma, systemic treatment may be required. Currently, systemic options are limited. There is some evidence that somatostatin analogs may slow down or stop progression of primary paragangliomas and their metastases [12, 13]. Somatostatin is a central regulator of neuroendocrine cell physiology. Somatostatin analogs have antisecretory and antiproliferative effects and have been shown to be effective in other neuroendocrine tumors [14]. Pheochromocytomas and paragangliomas may represent good targets for this therapeutic modality, as these tumors overexpress somatostatin receptors. The current patient was treated with the somatostatin analog lanreotide, and the disease has remained stable since, both at the primary and metastatic sites.

In conclusion, we present a patient with a known pathogenic *SDHAF2* variant, which resulted in multiple head and neck paragangliomas, a paraganglioma in the pancreas and paraganglioma metastases to the lung and spine. It remains unclear whether the lesion in the pancreas represents primary or metastatic disease. Even so, this report shows that, contrary to first reports on *SDHAF2*, abdominal and metastatic disease can be part of the *SDHAF2*-linked phenotype. This observation is an important addition to the clinical spectrum of *SDHAF2*-linked disease and has ramifications for *SDHAF2*-linked paraganglioma patients. They should be informed about this possibility, and like in carriers of other pathogenic SDH gene variants, whole body tumor surveillance seems warranted in pathogenic *SDHAF2* variant carriers too.

## Figures and Tables

**Table 1:** Overview of patient medical history.

Location	Age at diagnosis	Pathology	Treatment	Progression	Complaints
Bilateral carotic body tumors	14	Tumor resection	Surgical removal bilaterally at age 19	Right recurrence	Swelling neck
Bilateral vagal	27	Lacking	Active surveillance	Left vagal slowly progressing	Right vocal fold paresis
Jugulotympanic	34	Tumor resection	Partial surgical removal at age 50		Troublesome pulsatile tinnitus
Pancreas head	49	Cytology	Lanreotide	Stable	—
Paravertebral metastasis	49	Histology	Lanreotide	Stable	—
Lung metastasis	49	Lacking	Lanreotide	Stable	—

**Table 2:** Family history.

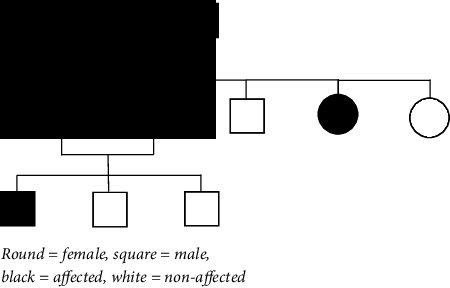

**Table 3:** Catecholamine value.

	Reference	At age 49	At age 50	At age 51	At age 52
Metanephrines (nmol/L)	0.07–0.33	0.10	—	—	—
Free metanephrines (pmol/L)	70–330	—	136	131	121
3-Methoxytyramine (nmol/L)	0.00–0.04	0.02	—	—	—
Free 3-methoxytyramine (pmol/L)	0–40	—	68 (H)	44 (H)	24

## Data Availability

The data supporting this case report are from previously reported studies and datasets, which have been cited. The processed data are available from the corresponding author upon request.
